# Association Between Homelessness and Mental Healthcare Utilization Among People With Addictions and Mental Health Problems: A Population-Based Study From Alberta Canada

**DOI:** 10.1177/07067437261462692

**Published:** 2026-07-16

**Authors:** Rebecca Barry, Geoffrey Messier, Anees Bahji, Mohammad Ziaul Islam Chowdhury, Gina Dimitropoulos, Sumantra Monty Ghosh, Julia Kirkham, Scott B Patten, Katherine Rittenbach, Faezehsadat Shahidi, David Tano, Valerie H Taylor, Dallas P Seitz

**Affiliations:** 1 Department of Psychiatry, 2129 University of Calgary, Calgary, Canada; 2 Hotchkiss Brain Institute, 2129 University of Calgary, Calgary, Canada; 3 Mathison Centre for Mental Health Research and Education, 2129 University of Calgary, Calgary, Canada; 4 Department of Electrical and Software Engineering, 2129 University of Calgary, Calgary, Canada; 5 Department of Community Health Sciences, 2129 University of Calgary, Calgary, Canada; 6 Department of Psychiatry, Cumming School of Medicine, University of Calgary, Calgary, Alberta, Canada; 7 Alberta Health Services, Calgary, Canada; 8 Faculty of Social Work, 2129 University of Calgary, Calgary, Canada; 9 Cumming School of Medicine, 2129 University of Calgary, Calgary, Canada

**Keywords:** homelessness, mental disorders, cohort, mortality, healthcare utilization, suicide attempt

## Abstract

**Objective:**

Addiction and mental health (AMH) disorders present significant psychosocial challenges among affected individuals. Previous research indicates a higher prevalence of AMH conditions among people experiencing homelessness (PEH). This study aims to determine the risk of mortality, suicide attempts, emergency department (ED) visits and hospitalizations among PEH with AMH conditions compared to individuals with AMH conditions who had not experienced homelessness in Alberta.

**Methods:**

This retrospective cohort study used linked administrative data from Alberta Health Services from April 1, 2013 to March 31, 2023. We included adults residing in Alberta and diagnosed with any AMH condition within 5 years before the index date of April 1, 2018. Homelessness in the year prior to the index date was identified using relevant codes in records representing hospitalizations and ED visits. We used Cox Proportional Hazards models to evaluate the risk of ED visits, hospitalizations, suicide attempts, and all-cause mortality for up to 5 years, adjusted for age and sex. We also conducted matched propensity score analyses.

**Results:**

Among the 622,614 individuals with AMH conditions, 3,390 (0.54%) had an indicator of homelessness. In age and sex-adjusted analyses, PEH were at greater risk for ED visits for both AMH (HR = 8·75, 95% CI [8.37–9.16]) and non-AMH (HR = 2.56, HR = 2.47–2.63) reasons, and at greater risk of hospitalizations for AMH (HR = 8.34, 95% CI [7.73–9.00]) and non-AMH (HR = 6.03, 95% CI [5.73–6.35]) reasons. PEH were at greater risk of suicide attempts (HR = 9.18, 95% CI [8.43–10.01]) and all-cause mortality (HR = 8.15, 95% CI [7.56–8.79]). Findings were attenuated but still significant in the propensity-matched analyses.

**Conclusions:**

PEH are at much greater risk of ED visits, hospitalizations, suicide attempts and all-cause mortality. Given this population's high risk of adverse outcomes, PEH will likely need more intensive mental and physical healthcare, coordinated with housing and social support services. Further research is needed on the implementation of interventions tailored to the needs of PEH.

## Introduction

Addictions and other mental health (AMH) conditions are associated with significant societal and economic burdens, contributing to increased healthcare utilization, and reduced productivity.^1^ Globally, people experiencing homelessness (PEH) are disproportionately affected by AMH conditions, with 67% diagnosed with an AMH condition.^2^ A high prevalence of substance use disorders (44%), major depression (19%), posttraumatic stress disorder (10.5%), schizophrenia (7%), and bipolar disorder (8%) has been identified among PEH.^2^

While it is known that PEH with AMH face greater comorbidity, significant barriers to accessing care, and other social challenges (e.g., stigmatization, unmet social needs, and difficulty securing basic needs)^3,4^ the long-term trajectory of AMH outcomes among PEH remains poorly understood. Several studies have found an increased risk of mortality among PEH with AMH.^5–7^ However, few studies have investigated the risk of increased healthcare utilization (e.g., ED visits, hospitalizations) and suicide attempts specifically among PEH with AMH conditions compared to those not experiencing homelessness with AMH conditions. From a public health perspective, identifying the risk of adverse outcomes is essential for guiding decision making and developing effective strategies to enhance quality of life, lower the risk of mortality and reduce healthcare costs.

In a previous study, we compared baseline characteristics between PEH and those not experiencing homelessness, among those with AMH.^8^ We found that PEH had greater healthcare utilization, more comorbid conditions, and a greater likelihood of almost all AMH conditions at baseline.^8^ Building on these findings, the current study aims to determine the risk of adverse outcomes among PEH during the following 5 years, while adjusting for these baseline differences. This study aims to determine the risk of mortality, suicide attempts, death by suicide, ED visits and hospitalizations among individuals with AMH conditions, comparing those who had recently experienced homelessness to those who had not. These findings will help inform outcome-specific interventions tailored to the needs of PEH with AMH. We hypothesize that homelessness will be a strong risk factor for outcomes including suicide attempts, increased healthcare utilization, all-cause mortality and death by suicide. This study may also highlight the urgency for early intervention for AMH conditions and the need for timely housing interventions such as Housing First.

## Methods

This cohort study utilizes linked data from April 1, 2013 until March 31, 2023. Baseline data (from April 1, 2013 to March 31, 2018) was extracted from the Alberta Health Services (AHS) Enterprise Data Warehouse with support provided by the Alberta Strategy for Patient Oriented Research Support Unit (AbSPORU), while follow-up data was extracted from the AHS Enterprise Data Warehouse by the authors.

### Data Sources

This analysis utilizes health services data from AHS, Alberta Health and related administrative data. These data sources include the Physician Claims database, the Canadian Institute for Health Information Discharge Abstract Database (CIHI-DAD), the Canadian Institute for Health Information National Ambulatory Care Reporting System (CIHI-NACRS), the Registry database, the Pampalon Material and Social Index Deprivation database, and Vital Statistics. These datasets were linked through unique encoded identifiers, based on scrambled personal health numbers.

### Inclusion Criteria

The details of the study cohort inclusion criteria, case definition of homelessness, and covariate definitions used in the current study have been previously described.^8^ To summarize, we included individuals diagnosed with an AMH condition within 5 years prior to the index date of April 1, 2018 living in Alberta, Canada. Individuals were aged 18 to 65 and alive on the index date. Individuals had to be included in the provincial health insurance registry on the index date, which provides near-complete coverage of the Alberta population eligible for publicly funded healthcare services. AMH conditions were identified based on either a hospitalization with a relevant AMH diagnosis or at least two relevant AMH diagnosis claims separated by at least 30 days within a 2-year time period. We categorized AMH disorders into 15 groups: mood disorders, anxiety disorders, substance use disorders (excluding tobacco or nicotine dependence), psychotic disorders, cognitive disorders, developmental disabilities, personality disorders, eating disorders, sexual disorders, attention-deficit/hyperactivity disorder, other childhood and developmental disorders, organic disorders, sleep disorders, somatic disorders and self-harm. We used any recorded code in DAD or claims, not limited to primary diagnoses. Details on all relevant AMH diagnosis codes are included in Appendix 1. Because inclusion required a documented AMH diagnosis, this study reflects a subset of PEH and non-PEH individuals already engaged in healthcare services.

### Case Definition for Homelessness

PEH over the past year were identified through hospitalizations or emergency department (ED) visits with ICD-10 diagnostic codes Z59.0 or Z59.1.^9^ The time period for the definition of homelessness was the year prior to the index date of April 1, 2018 with an additional 15 days before and after this time period, meeting the validated algorithm criteria for “annual homelessness” as defined by Richard et al.^9^ This definition of homelessness has a specificity of 99.9%, but a sensitivity ranging from 19% to 36%.^8^

### Baseline Variables

We examined several categories of variables potentially related to homelessness including demographics, psychiatric multimorbidity, general medical conditions, and healthcare utilization at baseline. All baseline characteristics were measured from April 1, 2013 to March 31, 2018.

**Demographics***:* We used demographic information including age, sex, and socioeconomic status. We used the Registry database, a population-based registry for the province of Alberta which includes data on individual's age and sex and eligibility for public health care insurance. We used the material and social Pampalon deprivation indices^10^ to define socioeconomic status. These are area-based measures of deprivation, based on census data that is aggregated at the level of dissemination areas. These are the smallest statistical units for which census data are normally released. They are intended to be relatively homogeneous in terms of socioeconomic conditions.

**General medical comorbidity and multimorbidity:** We use the Elixhauser comorbidity^11,12^; index, based on Physicians Claims data, CIHI-DAD and CIHI-NACRS to measure general medical comorbidities at baseline (e.g., congestive heart failure, hypertension, diabetes), excluding mental health–related comorbidities. We also examined the prevalence of traumatic brain injury. Overall, we examined 31 conditions.

**Healthcare utilization:** We defined healthcare usage over the 5 years prior to baseline (April 1, 2013 to March 31, 2018) using CIHI-NACRS for ED visits, the Physician Claims database for physician and psychiatrist visits, and CIHI-DAD for hospitalizations.

### Outcomes

Outcomes were measured from April 1, 2018 to March 31, 2023. The primary outcome was ED visits for AMH reasons, defined using CIHI-NACRS. Other outcomes of interest were AMH-related, non-AMH-related, or any hospitalization defined using DAD, and any ED visit or non-AMH ED visit using CIHI-NACRS. We examined suicide attempts using CIHI-NACRS using codes X60-X84, Y10-Y19, and Y28.^13^ We defined death by suicide using codes for the underlying cause of deaths (E950-E959, X60-X84, Y10-Y19, and Y28) using the Vital Statistics database. We examined all-cause mortality using the death date in Vital Statistics.

### Statistical Analysis

Survival analysis was performed using Cox proportional hazards models to evaluate the associations between homelessness and each outcome. First, we assessed the time to the occurrence of the first of each outcome (i.e., ED visit, hospitalization) for PEH compared to those not experiencing recent homelessness, adjusting for age and sex. Individuals were censored in the event of death and were followed for a maximum of 5 years following the index date (April 1, 2018 to March 31, 2023). We also conducted a sensitivity analysis where only those with a DAD or NACRS indicator during the year prior (plus 15 days on each end) were included in the study to mirror the homelessness definition.

Next, a propensity score matched analysis was performed with a 1:1 match of PEH to comparable individuals who did not experience homelessness. All baseline characteristics of the cohort were included in the model to create the propensity score match. We matched on age (1 year) and sex, to allow for interaction terms to be examined for age and sex. For the remaining variables included in the propensity score, we matched based on an ideal caliper width of 0.2 times the standard deviation of the logit of the propensity score.^14^ We then examined standardized differences between the matched groups across all matching variables. Following matching, Cox proportional hazards models were stratified by matched pairs to account for the matched design. After matching, we also considered interactions by age and sex in this analysis to determine if the associations between homelessness and outcomes varied across age groups or by sex.

All Cox proportional hazards models included death as a competing risk because deaths were more likely among those experiencing homelessness and also excludes the possibility of other outcomes. Analyses were completed using SAS version 9.4^15^ using two-sided *P*-values of .05 as the threshold for statistical significance.

### Ethics

This study was reviewed and approved by the Conjoint Health Research Ethics Board at the University of Calgary (REB21-0070).

## Results

### Description of Study Population

Among the 622,614 individuals with AMH conditions, 3,390 (0.54%) met the criteria for homelessness. Baseline differences among the full study population are thoroughly described in our previous paper.^8^ Notably, in the 5 years prior to index, PEH were more likely to be diagnosed with all AMH disorders other than sleep disorders and sexual disorders. PEH were more likely to be diagnosed with most Elixhauser chronic health conditions including chronic obstructive pulmonary disorder, hypertension, and congestive heart failure. They also had higher healthcare utilization, including prior ED visits, hospitalizations and psychiatrist visits.

### Association Between Homelessness and Outcomes in Full Cohort

Findings indicate that PEH with AMH have a significantly greater risk of AMH-related ED visits (HR = 8.75, 95% CI = 8.37–9.16), and to a lesser degree, non-AMH-related ED visits (HR = 2.56, HR = 2.47–2.63) compared to people not experiencing homelessness with AMH conditions (Table 1). PEH with AMH also have a greater risk of being hospitalized for AMH reasons (HR = 8.34, 95% CI = 7.73–9.00) and non-AMH reasons (HR = 6.03, 95% CI = 5.73–6.35). Finally, they are at greater risk of suicide attempts (HR = 9.18, 95% CI = 8.43–10.01), death by suicide (HR = 2.20, 95% CI = 1.30–3.74) and all-cause mortality (HR = 8.15, 95% CI = 7.56–8.79).

**Table 1.** Survival Analysis Results Using Cox Proportional Hazards Models (Full Cohort).

Outcome	Experiencing Homelessness (*N* = 3,390)	Not experiencing Homelessness (*N* = 659,224)	Hazard Ratio [95% CI]*
Emergency department visits			
Emergency department visit for mental health reasons	2,509 (74.0)	101,650 (15.4)	8.75 [8.37, 9.16]
Emergency department visit for non-mental health reasons	3,157 (93.1)	524, 879 (79.6)	2.56 [2.47, 2.63]
Any emergency department visit	3,220 (95.0)	531,730 (80.7)	2.92 [2.79, 3.07]
Hospitalizations			
Hospitalization for mental health reasons	947 (27.9)	23,800 (3.6)	8.34 [7.73, 9.00]
Hospitalization for nonmental health reasons	1,859 (54.8)	88,687 (13.5)	6.03 [5.73, 6.35]
Any hospitalization	2,287 (67.5)	104, 896 (15.9)	7.17 [6.83, 7.54]
Suicide attempts and death			
Suicide attempt	587 (16.7)	12,563 (1.9)	9.18 [8.43, 10.01]
Completed suicide	14 (0.4)	960 (0.2)	2.20 [1.30, 3.74]
All-cause mortality	715 (21.1)	18,002 (2.7)	8.15 [7.56, 8.79]

*adjusted for age group and sex.

After including only those with a hospitalization or ED visit in the year prior to index (plus 15 days before and after), the population of those not experiencing homelessness was reduced from 659,224 to 373,537. The comparisons between this population and those experiencing homelessness, as well as the hazard ratios depicting the risk of adverse outcomes are shown in Table 2. The findings, although attenuated, are similar to the findings using the entire study population.

**Table 2.** Survival Analysis Results Using Cox Proportional Hazards Models (Full Cohort, Sensitivity Analysis).

Outcome	Experiencing Homelessness (*N* = 3,390)	Not Experiencing Homelessness (*N* = 373,537)	Hazard Ratio [95% CI]*
Emergency department visits			
Emergency department visit for mental health reasons	2,509 (74.0)	77, 638 (20.8)	5.73 [5.48, 5.99]
Emergency department visit for nonmental health reasons	3,157 (93.1)	335,386 (89.8)	1.65 [1.60, 1.71]
Any emergency department visit	3,220 (95.0)	339, 043 (90.8)	1.85 [1.77, 1.93]
Hospitalizations			
Hospitalization for mental health reasons	947 (27.9)	17,943 (4.8)	5.93 [5.51, 6.37]
Hospitalization for nonmental health reasons	1,859 (54.8)	66,792 (17.9)	4.29 [4.08, 4.51]
Any hospitalization	2,287 (67.5)	78,389 (21.0)	5.07 [4.83, 5.31]
Suicide attempts and death			
Suicide attempt	567 (16.7)	10,035 (2.7)	6.14 [5.63, 6.70]
Suicide	14 (0.4)	673 (0.2)	1.71 [1.00, 2.91]
All-cause mortality	715 (21.1)	14,007 (3.8)	5.84 [5.42, 6.30]

### Propensity Score Matched Analysis

After matching, 3,352 (98.9%) of PEH with AMH were matched to similar individuals not experiencing homelessness. Standardized differences in baseline characteristics between groups post-match are shown in Table 3, and all standardized differences are less than 0.1.

**Table 3.** Standardized Differences in Baseline Characteristics After Propensity Score Matching.

Baseline Characteristic	Standardized Difference
Age	0
Sex	0
Pampalon index: social	−0.006
Pampalon index: material	0.011
Anxiety disorder	0.043
Mood disorder	0.051
Substance use disorder	−0.029
Psychotic disorder	0.004
Cognitive disorder	0.021
Developmental disability	−0.020
Personality disorder	0.020
Eating disorder	0.029
Sexual disorder	−0.011
Other childhood and developmental disorders	0.003
Attention deficit hyperactivity disorder	0.016
Organic disorders	0.027
Sleep disorders	0.013
Somatic symptoms and related disorders	−0.029
Self-harm	0.042
Any psychiatrist visit (5 year)	0.014
Any ED mental health visit (5 year)	−0.001
Any ED nonmental health visit (5 year)	−0.005
Any mental health hospitalization (5 year)	0.025
Any nonmental health hospitalization (5 year)	−0.006
Congestive heart failure	0.003
Cardiac arrhythmia	0.041
Hypertension, uncomplicated	0.022
Hypertension, complicated	0.024
Other neurological disorders	0.039
Chronic obstructive pulmonary disease	0.026
Diabetes, uncomplicated	0.035
Diabetes, complicated	0.025
Liver disease	0.035
Peptic ulcer disease	0.014
AIDS/HIV	0.029
Seizure disorder	0.058
Dyslipidemia	−0.002
Anemia	0.002
Traumatic brain injury/head injury	0.0074
Parkinson's disease	0.021
Peripheral vascular disease	−0.015
Coronary heart disease	0.016
Valvular disease	0.009
Stroke	0.011
Renal failure	0.008
Solid tumor	0.025
Paralysis	−0.008
Weight loss	0.034
Metastatic cancer	0.003
Lymphoma	0.013
Obesity	0.029
Fluid and electrolyte disorders	0.044
Hypothyroidism	0.035
Liver disease	0.035
Rheumatoid arthritis	0.050

Findings from the survival analyses stratified by matched pairs indicate that PEH with AMH had a significantly greater risk of AMH-related ED visits (HR = 1.98, 95% CI = 1.87–2.10) and non-AMH-related ED visits (HR = 1.57, 95% CI = 1.49–1.65) (Table 4). They have a greater risk of hospitalizations for AMH reasons (HR = 1.56, 95% CI = 1.45–1.69) and non-AMH reasons (HR = 1.80, 95% CI = 1.69–1.92)). Finally, PEH with AMH are at greater risk of suicide attempts (HR = 1.50, 95% CI = 1.36–1.65) and all-cause mortality (HR = 1.67, 95% CI = 1.45, 1.89). We were unable to measure the risk of suicide, as too few deaths due to suicide were recorded.

**Table 4.** Survival Analysis Results Using Cox Proportional Hazards Models (Propensity-Matched Cohort).

Outcome	Experiencing Homelessness (*N* = 3,352)	Not experiencing Homelessness (*N* = 3,352)	Hazard Ratio [95% CI]
Emergency department visits			
Emergency department visit for mental health reasons*	2,474 (73.8)	1,805 (53.9)	1.98 [1.87, 2.10]
Emergency department visit for nonmental health reasons	3,119 (93.1)	2,905 (86.7)	1.57 [1.49, 1.65]
Any emergency department visit*	3,182 (94.9)	2,972 (88.7)	1.65 [1.57, 1.73]
Hospitalizations			
Hospitalization for mental health reasons	932 (27.8)	659 (19.7)	1.56 [1.45, 1.69]
Hospitalization for nonmental health reasons*	1,827 (54.5)	1,235 (36.8)	1.80 [1.69, 1.92]
Any hospitalization	2,252 (67.2)	1,570 (46.8)	1.91 [1.80, 2.02]
Suicide attempts and death			
Suicide attempt*	556 (16.6)	381 (11.4)	1.50 [1.36, 1.65]
All-cause mortality	702 (20.9)	447 (13.3)	1.67 [1.45, 1.89]

No interaction terms by sex were significant. However, several interaction terms by age were significant. Where significant, we included the stratified results in Table 5. All stratified findings indicate the same direction of association, but the risk between homelessness and several outcomes was greater among younger people compared to older people. A minor observation is that the association between homelessness and suicide attempts was not statistically significant among individuals aged 45–56, although the confidence interval (HR = 1.17, 95% CI = 0.91–1.49) suggests a possible effect and may reflect limited power in this subgroup.

**Table 5.** Survival Analysis Results Using Cox Proportional Hazards Models (Propensity-Matched Cohort) Stratified by Age for Significant Interaction Terms

Outcome	Hazard Ratio [95% CI]
Emergency department visits	
Emergency department visit for mental health reasons (overall)	1.98 [1.87, 2.10]
Ages 18–25	2.35 [2.01, 2.75]
Ages 26–35	2.00 [1.80, 2.23]
Ages 36–45	2.03 [1.81, 2.28]
Ages 46–55	1.69 [1.49, 1.91]
Ages 56–65	1.94 [1.63, 2.32]
Any emergency department visit (overall)	1.65 [1.57, 1.73]
Ages 18–25	1.75 [1.53, 2.00]
Ages 26–35	1.70 [1.54, 1.87]
Ages 36–45	1.65 [1.49, 1.83]
Ages 46–55	1.67 [1.50, 1.87]
Ages 56–65	1.41 [1.23, 1.62]
Hospitalizations	
Hospitalization for nonmental health reasons (overall)	1.80 [1.69, 1.92]
Ages 18–25	2.15 [1.77, 2.61]
Ages 26–35	1.90 [1.68, 2.16]
Ages 36–45	1.64 [1.45, 1.86]
Ages 46–55	1.75 [1.54, 1.99]
Ages 56–65	1.76 [1.50, 2.07]
Suicide attempts and death	
Suicide attempt (overall)	1.50 [1.36, 1.65]
Ages 18–25	1.64 [1.31, 2.06]
Ages 26–35	1.60 [1.35, 1.91]
Ages 36–45	1.48 [1.21, 1.82]
Ages 46–55	1.17 [0.91, 1.49]
Ages 56–65	1.50 [1.02, 2.21]

## Discussion

Overall findings indicate that PEH with AMH conditions have a greater risk of ED visits (both AMH and non-AMH-related) and hospitalizations (both AMH and non-AMH-related) compared to other individuals not experiencing homelessness with AMH conditions. They also have a greater risk of suicide attempts, death by suicide and all-cause mortality. These results were persistent across both the full population and propensity-matched pairs. They were also persistent in the sensitivity analysis restricting the comparison group to only those with prior year ED visits or hospitalizations, although the associations were attenuated in the propensity score matched analysis.

While few studies have investigated the risk of ED visits specifically among PEH with AMH conditions compared to those not experiencing homelessness with AMH conditions, a recent systematic review found that PEH accounted for 0.4% to 19.6% of all ED visits across included studies and made an average of 0.7 to 5.8 ED visits per year.^16^ Lam et al. found that among PEH with MH visiting the ED, 31.1% revisited the ED and 3.7% were rehospitalized within 30 days.^17^ Comparatively, among those with MH not experiencing homelessness, 25.2% revisited the ED and 2.6% readmitted to the hospital.^17^ Laliberté et al.^18^ found that among people discharged from psychiatric hospitals, PEH were significantly more likely to have a readmission or ED visit within 30 days. Among Medicaid patients, Amato et al. found that PEH were 10.6 times more likely to be readmitted to the ED within a year.^19^ Among individuals experiencing homelessness, Wiens et al.^20^ found that a higher proportion of those with an AMH diagnosis accessed inpatient services or had an ED visit at 1 year follow-up. Overall, our findings are in agreement with other studies suggesting that PEH with AMH diagnoses have greater ED utilization compared to individuals with AMH diagnoses not experiencing homelessness.

Our findings are consistent with other studies that have examined the risk of hospitalizations among PEH with AMH over time. Russell et al.^21^ examined 60 youth attending a specialist mental health service over 8 years and found that those experiencing homelessness were 11 times more likely to be readmitted to a mental health inpatient ward, compared to our findings of an 8 times increase in AMH-related hospitalizations. Moss et al. found that among people attending an ED, the risk of hospital admissions over the following 4 years was higher among PEH (IRR=1.79, 95% CI = 1.69–1.90).^22^

While it is challenging to find similar studies examining the risk of suicide attempts among PEH with AMH, other studies have also indicated that PEH have a higher prevalence of suicide. Ayano et al. found the lifetime prevalence of suicide attempts among PEH to be 29% after systematically reviewing the literature.^23^ Bommersbach et al. found that among PEH, 21% reported a suicide attempt in the past year, compared to 5.8% among those who experienced homelessness before the past year, and 6.3% of those who had never experienced homelessness.^24^ Our findings are also consistent with studies examining the risk of mortality over time. An Australian cohort study found that PEH had a greater risk of mortality over 15 years (HR = 1.76, 95% CI = 1.49–2.08), among adults attending an ED,^6^ which is similar to our propensity-matched findings (HR = 1.67, 95% CI = 1.45–1.89). Several other studies have also found a greater risk of mortality among PEH.^5,7^

Given the elevated risk of hospitalizations, ED visits, suicide attempts and mortality among PEH with AMH, implementation of effective interventions is needed to help mitigate adverse outcomes and reduce overall healthcare costs. Housing first strategies where housing is provided without preconditions and later incorporates other supports, and critical time interventions where individuals are connected to supports during transitional periods, have been shown to improve housing retention, decrease psychiatric hospitalizations, and potentially improve AMH symptom severity.^25–29^ Housing first strategies implemented among PEH with AMH have also been shown to significantly reduce societal costs, with economic analyses indicating significant cost savings potentially outweighing the cost of housing.^30^ Assertive community treatment (ACT), which involves a multidisciplinary team providing intensive community-based mental health services, has also shown effectiveness in reducing both AMH-related symptom severity and homelessness among people with AMH.^31^ Housing First combined with ACT has been shown to reduce days in hospital and ED visits.^32^ However, as our findings indicate that PEH with AMH are at greater risk of ED visits and hospitalizations, future research might also investigate how strategies typically used to address high users of healthcare services might be applied among healthcare users with a recent history of homelessness. For example, real-time electronic notifications that flag patients at high risk of frequent utilization upon ED registration can prompt initiation of outreach by care coordinators or case managers to provide tailored discharge planning, connection to outpatient services or arranging follow-up after discharge, potentially lowering healthcare utilization and costs.^33,34^ Case management, an integrated approach across community and hospital settings where multidisciplinary teams coordinate health services on behalf of the patient, has not only been shown to improve clinical outcomes but also reduce homelessness.^35^

Our observational study has several strengths. First, we implemented multiple methods and a sensitivity analysis to examine the risk of adverse outcomes and although results were attenuated after matching or restricting the sample of those not experiencing homelessness, the overall conclusions were consistent. Additionally, this retrospective cohort study can examine temporal relationships between homelessness and outcomes and can examine multiple outcomes. This study also uses competing risks to account for mortality. This study also has a large sample size, with the power to detect smaller differences. Almost all PEH were able to be propensity score matched with a similar individual not experiencing homelessness, with 98.9% having a match. Finally, this study contributes to the literature as few studies have examined the relationship between homelessness and these outcomes over time with a comparison population.

This study also has limitations that need to be considered in interpreting these results. First, the cohort includes only individuals with documented AMH diagnoses based on administrative data, and therefore findings cannot be generalized to PEH without a diagnosed or documented AMH condition. Second, there may be unmeasured barriers to accessing care that could influence whether ED visits and hospitalizations were captured. However, PEH are known to experience greater barriers to accessing care^3,4^; so this would likely bias findings toward the null. Third, there is a possibility of unmeasured baseline characteristics such as disease severity that were not included in the propensity score, potentially leading to residual confounding. Fourth, we were unable to examine suicide as an outcome in the propensity-matched analyses due to power, which may reflect the relatively low number of deaths by suicide, as well as potential underreporting or misclassification in vital statistics. Fifth, the relationship between homelessness and the observed outcomes may be temporal, but that does not exclude the possibility that other instances of the outcomes (aside from mortality) may also have occurred prior to the onset of homelessness. For example, several studies have suggested that suicidality may occur prior to homelessness.^24,36^ Finally, while the homelessness definition used^9^ has a very high specificity (∼99.9%), its low sensitivity (19–36%) may systematically exclude individuals who experience homelessness but do not present to the hospital or are not coded as such. This likely results in an underrepresentation of individuals with lower acuity or limited healthcare access, potentially biasing results toward a sicker subset of PEH. Future research should explore these differences to better understand potential risk gradients within the broader PEH population.

Overall, our findings persistently show an increased risk of healthcare usage and adverse outcomes including suicide attempts and mortality among PEH over time. Frequently cited approaches such as housing first, critical time, and ACT may help reduce the severity of AMH and improve housing retention, but evidence-based interventions have not yet been implemented at scale. However, given the high risk of ED visits and hospitalizations, strategies typically used to improve outcomes among individuals with high healthcare utilization should be explored. Interventions tailored to the needs of PEH are critically needed to prevent elevated risk of premature death, suicide attempts, ED visits, and hospitalizations.

## Supplemental Material

sj-docx-1-cpa-10.1177_07067437261462692 - Supplemental material for Association Between Homelessness and Mental Healthcare Utilization Among People With Addictions and Mental Health Problems: A Population-Based Study From Alberta Canada Supplemental material, sj-docx-1-cpa-10.1177_07067437261462692 for Association Between Homelessness and Mental Healthcare Utilization Among People With Addictions and Mental Health Problems: A Population-Based Study From Alberta Canada by Rebecca Barry, Geoffrey Messier, Anees Bahji, Mohammad Ziaul Islam Chowdhury, Gina Dimitropoulos, Sumantra Monty Ghosh, Julia Kirkham, Scott B Patten, Katherine Rittenbach, Faezehsadat Shahidi, David Tano, Valerie H Taylor and Dallas P Seitz in The Canadian Journal of Psychiatry
