# Supplementary material for: Association Between Homelessness and Mental Healthcare Utilization Among People With Addictions and Mental Health Problems: A Population-Based Study From Alberta Canada
Source: Can J Psychiatry. 2026 Jul 16:07067437261462692. Online ahead of print. doi: 10.1177/07067437261462692 (PMC13375854; doi:10.1177/07067437261462692)
Supplement: sj-docx-1-cpa-10.1177_07067437261462692 - Supplemental material for Association Between Homelessness and Mental Healthcare Utilization Among People With Addictions and Mental Health Problems: A Population-Based Study From Alberta Canada [file sj-docx-1-cpa-10.1177_07067437261462692.docx]

Appendix 1: AMH diagnosis codes

|  | **AMH Case Definitions** |
| --- | --- |
| **AMH Conditions** | MUST have one or more of the following to be included in the cohort: Substance use disorder, Mood disorder, Anxiety disorder, Psychotic disorder, Cognitive Disorders, Developmental disability, Personality disorder, Conduct, Disorder, Eating disorder, Sexual disorder, ADHD, Organic disorder, Sleep disorder, Somatic symptoms and related disorders, and/or deliberate self-harm |
| **Substance Use Disorder** | DAD, any hospitalization: F10.X—F19.X (except F17), F55.X, F63.X  OR  Physician Claims: 2 or more claims at least 30 days apart in 2-year period with diagnosis: 291, 292,303, 304, 305 (except 305.1) |
| **Mood disorder** | DAD: F30.X, F31.X, F32.X, F33.X, F34.X, F38.X, F39.X, F53.0  OR  2 physicians claims at least 30 days apart within a 2 year period with one or more of the following codes:  Physician Claims: 296, 311 |
| **Anxiety disorder** | DAD: F40.X, F41.X, F42.X, F43.X, F48.8, F48.9  OR  2 physicians claims at least 30 days apart within a 2 year period with one or more of the following codes:  Physician Claims: 300, 308, 309 |
| **Psychotic disorder** | DAD: F06.0, F06.1, F06.2, F06.0-2, F20.X, F22.X, F23.X, F24.X, F25.X, F26.X, F27.X, F28.X, F29.X, F22-F29, F53.1  OR  2 physicians claims at least 30 days apart within a 2 year period with one or more of the following codes:  Physician Claims: 295, 297, 298 |
| **Cognitive disorders** | DAD: F00X, F01.X, F02.X, F03.X G30.X  OR  2 physicians claims at least 30 days apart within a 2 year period with one or more of the following codes:  Physician Claims: 290, 331  OR  Prescription of dementia medication: Pharmacy Information Network: DINS for Donepezil, Galantamine, Rivastigmine or Memantine (Excel file attached) |
| **Developmental disability** | DAD: F70.0, F70.1, F70.8, F70.9, F71.0, F71.1, F71.8, F71.9, F72.0, F72.1, F72.8, F72.9, F73.0, F73.1, F73.8, F73.9, F78.0, F78.1, F78.8, F78.9, F79.0, F79.1, F79.8, F79.9, F84.0, F84.1, F84.3, F84.4, F84.5, F84.8, F84.9  OR  2 physicians claims at least 30 days apart within a 2 year period with one or more of the following codes:  Physician Claims: 299, 317, 318, 319 |
| **Personality disorders** | DAD: F21.X, F60.X, F61.X, F62.X, F68.1, F68.8, F69.X, F91.X  OR  2 physicians claims at least 30 days apart within a 2 year period with one or more of the following codes:  Physician Claims: 301, 312 |
| **Eating disorders** | DAD: F50  OR  2 physicians claims at least 30 days apart within a 2 year period with one or more of the following codes:  Physician Claims: 307.1, 307.51, 307.50 |
| **Sexual disorders** | DAD: F52.X, F64.X, F65.X  OR  2 physicians claims at least 30 days apart within a 2 year period with one or more of the following codes:  Physician Claims: 302 |
| **Other childhood and developmental disorders (not captured in first definition)** | DAD: F80.X, F81.X, F82.X, F83.X, F85.X, F86.X, F87.X, F88.X, F89.X. F92.X, F93.X, F94.X, F95.X, F96.X, F97.X, F98.X, F99.X  OR  2 physicians claims at least 30 days apart within a 2 year period with one or more of the following codes:  Physician Claims: 307.0, 307.2, 307.3, 307.52, 307.53, 307.54, 307.59, 307.6, 307.7, 307.8, 307.9, 313, 315. |
| **ADHD** | DAD: F90  OR  2 physicians claims at least 30 days apart within a 2 year period with one or more of the following codes:  Physician Claims: 314 |
| **Organic disorders** | DAD: F06.X, F07.X, F09.X, F59.X  OR  2 physicians claims at least 30 days apart within a 2 year period with one or more of the following codes:  Physician Claims: 293, 294, 310 |
| **Sleep disorders** | DAD: F51  OR  2 physicians claims at least 30 days apart within a 2 year period with one or more of the following codes:  Physician Claims: 307.4 |
| **Somatic symptoms and Related Disorders** | DAD: F44.X, F45.X, F48.0, F48.1, F54.X, F68.0  OR  2 physicians claims at least 30 days apart within a 2 year period with one or more of the following codes:  Physician Claims: 306, 316 |
| **Deliberate self-harm** | DAD: X60-X84, Y10-Y19, Y28 when DX10CODE1 is not equal to F06-F99 |
